# Adherence to treatment guidelines: the association between stroke risk stratified comparing CHADS_2_ and CHA_2_DS_2_-VASc score levels and warfarin prescription for adult patients with atrial fibrillation

**DOI:** 10.1186/s12913-017-2025-6

**Published:** 2017-02-11

**Authors:** Scott A. Chapman, Catherine A. St Hill, Meg M. Little, Michael T. Swanoski, Shellina R. Scheiner, Kenric B. Ware, M. Nawal Lutfiyya

**Affiliations:** 1 Department of Experimental and Clinical Pharmacology, College of Pharmacy, University of Minnesota, Minneapolis, MN USA; 2 Department of Pharmacy Practice and Pharmaceutical Sciences, College of Pharmacy, University of Minnesota, Minneapolis, MN USA; 3 Department of Pharmacy Practice, College of Pharmacy, South University, Columbia, SC 29203 USA; 4 National Center for Interprofessional Education and Practice, R685 Children’s Rehabilitation Center, University of Minnesota, 426 Church Street SE, Minneapolis, MN 55455 USA

**Keywords:** Warfarin, CHADS_2_, CHA_2_DS_2_-VASc, Atrial fibrillation, Stroke, Guideline Adherence, Anti-coagulation, Interprofessional health care teams

## Abstract

**Background:**

Ischemic stroke is a risk associated with atrial fibrillation (AF) and is estimated to occur five times more often in afflicted patients than in those without AF. Anti-thrombotic therapy is recommended for the prevention of ischemic stroke. Risk stratification tools, such as the CHADS_2_, and more recently the CHA_2_DS_2_-VASc, for predicting stroke in patients with AF have been developed to determine the level of stroke risk and assist clinicians in the selection of antithrombotic therapy. Warfarin, for stroke prevention in AF, is the most commonly prescribed anticoagulant in North America. The purpose of this study was to examine the utility of using the CHADS_2_ score levels (low and high) in contrast to the CHA_2_DS_2_-VASc when examining the outcome of warfarin prescriptions for adult patients with AF. The CHA_2_DS_2_-VASc tool was not widely used in 2010, when the data analyzed were collected. It has only been since 2014 that CHA_2_DS_2_-VASc criteria has been recommended to guide anticoagulant treatment in updated AF treatment guidelines.

**Methods:**

Bivariate and multivariate data analysis strategies were used to analyze 2010 National Ambulatory Care Survey (NAMCS) data. NAMCS is designed to collect data on the use and provision of ambulatory care services nationwide. The study population for this research was US adults with a diagnosis of AF. Warfarin prescription was the dependent variable for this study. The study population was 7,669,844 AF patients.

**Results:**

Bivariate analysis revealed that of those AF patients with a high CHADS_2_ score, 25.1% had received a warfarin prescription and 18.8 for those with a high CHA_2_DS_2_-VASc score. Logistic regression analysis yielded that patients with AF had higher odds of having a warfarin prescription if they had a high CHADS_2_ score, were Caucasian, lived in a zip code where < 20% of the population had a university education, and lived in a zip code where < 10% of the population were living in households with incomes below the federal poverty level. Further, the analysis yielded that patients with AF had lesser odds of having a warfarin prescription if they were ≥ 65 years of age, female, or had health insurance.

**Conclusions:**

Overall, warfarin appears to be under-prescribed for patients with AF regardless of the risk stratification system used. Based on the key findings of our study opportunities for interventions are present to improve guideline adherence in alignment with risk stratification for stroke prevention. Interprofessional health care teams can provide improved medical management of stroke prevention for patients with AF. These interprofessional health care teams should be constituted of primary care providers (physicians, physician assistants, and nurse practitioners), nurses (RN, LPN), and pharmacists (PharmD, RPh).

## Background

Atrial fibrillation (AF), the most common sustained form of cardiac arrhythmia, is characterized by disorganized electrical activity in the atria accompanied by an irregular ventricular response that is usually rapid [1]. In 2010, there were 1.2 million new diagnoses of AF in the United States (US). During that same year, the prevalence of AF was estimated at 5.2 million in the US, and projected to increase to 12.1 million individuals by 2030 [2]. AF is an age prevalent condition. Estimates of the prevalence range from 0.1% of individuals less than 55 years of age to 9.0% of individuals over 80 years of age, with 3.8% of individuals 60 years and older having AF [3].

Stroke poses a significant health burden in the US and therefore prevention is warranted. In addition to the years of life lost (mortality) and disability (morbidity) the direct and indirect costs of stroke as part of the disease burden are considerable [4–7]. The projected total estimated cost of stroke will be at least $300 billion or more by 2050 [8, 9].

This significant cost for stroke underscores the importance of stroke prevention efforts. Treatment also contributes to the cost burden of stroke. Casciano, et al., [10] using prescription claims data as a proxy for prescription adherence, examined the cost burden associated with sub-optimal warfarin treatment and found that healthcare costs (fewer inpatient and hospital visits, and hospital days) were 13% lower for people with AF who were prescribed warfarin and were taking the drug.

Ischemic stroke is an important risk associated with AF and is estimated to occur five times more often in afflicted patients than those without AF [4]. Fifteen percent of all strokes are associated with AF [5]. Because of this risk, antithrombotic therapy is recommended for the prevention of ischemic stroke. In a pooled analysis of five randomized controlled trials evaluating stroke risk and antithrombotic therapy, the average stroke rate in patients not receiving antithrombotic therapy was found to be 4.5% per year [6]. Patients with AF who suffer stroke have an increased risk for: increased stroke severity, 30 day mortality, and higher rates of 1 year recurrence of stroke compared to stroke sufferers without AF [7].

Risk stratification tools for predicting stroke in patients with AF have been developed to determine the level of stroke risk and assist clinicians in the selection of antithrombotic therapy. The risk of stroke (reported as an adjusted stroke rate per 100 person years) is 1.9 (95% CI, 1.2–3.0) for a CHADS2 score of 0, and increases to 18.2 (95% CI, 10.5–27.4) for a CHADS_2_ score of 6 [11]. The CHA_2_DS_2_-VASc score was established in 2010 [12] as a refinement over the CHADS_2_ score in the predictive value of stroke [13]. Even though CHA_2_DS_2_-VASc was not used widely at the time the data were collected, we examined CHA_2_DS_2_-VASc criteria in relation to our data. We compared CHADS_2_ to CHA_2_DS_2_-VASc as the latter is now recommended in the AF treatment guidelines updated in 2014.

Although, in October 2010, dabigatran was approved by the FDA as the first non-vitamin K antagonist oral anticoagulant for prescription in the US, warfarin was approved much earlier in 1954 and remains the most commonly prescribed anticoagulant in North America, including its use for stroke prevention in AF [14, 15]. A meta-analysis including six trials comparing warfarin to placebo or no treatment, found that warfarin reduced ischemic stroke events by 67% [16]. Comparing CHADS_2_ and CHA_2_DS_2_-VASc as risk stratification strategies is an important consideration in prescribing warfarin therapy. CHA_2_DS_2_-VASc in contrast to CHADS_2_ more accurately identifies patients at low risk for ischemic stroke and moves more patients from the intermediate level to the high risk level. The importance should not be underestimated since doing so reduces the ambiguity of identifying those patients who actually need anti-thrombotic therapy [17].

Studies in the US examining patterns in anticoagulation for AF have shown an increase in the overall trend of warfarin use in the past several years [18–20]. Yet, warfarin appears to be under-prescribed [21, 22]. To assess the prescription patterns for warfarin as determined by CHAD_2_ versus CHA_2_DS_2_-VASc criteria, we analyzed data from the 2010 NAMCS, a large publically available database of patient records that are weighted to be representative of the US population. The large sample size and weighted data allow for generalizable results. At least one earlier study from 1996 [23] also examined NAMCS data. However, those data from multiple years, are decades old and were collected before either CHADS_2_ or CHA_2_DS_2_-VASc were developed. The purpose of this study was to examine the association between CHADS_2_ score levels (low and high) and warfarin prescriptions for adult patients with AF. We evaluated the prevalence of adherence to antithrombotic therapy for AF treatment guidelines that were in place when the data were collected [24] and compared these to the descriptive results of using the CHA_2_DS_2_-VASc system for risk assessment.

## Methods

Both bivariate and multivariate analytic approaches were used to examine 2010 NAMCS data in order to answer the research question. The NAMCS was designed and is used to collect data on the actual use and provision of ambulatory care services throughout the US. A national sample of ambulatory care visits are surveyed in order to collect the data. A complex four-stage probability sampling design is employed in the data collection process. A description of the sampling strategy is discussed elsewhere [25]. Because they were the most recently available data at the onset of this study, the 2010 NAMCS data were used. These data are weighted, by the survey designers, to be nationally representative of patient health records.

As recommended by the Center for Disease Control and Prevention’s (CDC) National Center for Health Statistics (NCHS), all analyses were performed on weighted data. The weighting, as calculated, uses the most recently available census data to provide a stratified representation of the nation’s primary care patient population. Only weighted data are reported in the results.

The *Patient Record Form* is the survey instrument for data abstraction from the medical record. The NAMCS patient record form is completed by ambulatory care clinic staff for a systematic random sample of patient visits during a randomly assigned 1-week reporting period. The data obtained include demographic characteristics of patients, expected source(s) of payment, patients’ complaints, diagnoses, diagnostic/screening services, procedures, medication therapy, disposition, types of providers seen, causes of injury, and certain characteristics of the clinic facility, such as geographic region and metropolitan status.

For this research project, the study population was US adults with a diagnosis of AF. The ICD-9 code for AF is 427.31. The covariates or independent variables for this research were: CHADS_2_ score levels (low and high), sex (male/female), race (Caucasian/Non-Caucasian), geographic locale (rural/non-rural), education attainment in a patient’s zip code (<20% of adults with a university degree/≥ 20% of adults with a university degree), poverty level in patient’s zip code (<10%/≥ 10%), health insurance status (insured/noninsured), and primary health care provider (HCP) seen (yes/no). Patient age was also a covariate in this study. For some of the analyses performed, CHADS_2_ age ranges are reported (<75 years/≥75 years), in other analyses three age-ranges were included (18–39, 40–64, and ≥ 65). For the logistic regression analysis performed the two age ranges used were 40–64 years and ≥ 65 years. All of the study covariates were recoded from their original configuration for analyses. Re-coding entailed either collapsing categories and/or removing unknown responses.

CHADS_2_, one of the study covariates or independent variables, is a clinical prediction tool for estimating the risk of stroke for patients with non-valvular AF. AF is associated with thromboembolic stroke. A CHADS_2_ score ranges from 0 to 6 and is computed from the following variables: congestive heart failure (1 point), hypertension (1 point), age ≥75 years (1 point), diabetes (1 point), and prior stroke or transient ischemic attack (TIA) (2 points). Patients with a score ranging from 0 to 1 were coded as having a low CHADS_2_ score and those with a score ≥ 2 were coded as having a high CHADS_2_ score. The stroke/TIA variable was computed from six, three digit ICD-9 codes for stroke (434.00, 434.01, 434.10, 434.11, 434.90, 434.91) and six, three digit ICD-9 codes for TIA (435.0, 435.1, 435.2, 435.3, 435.8, 435.9). Those patients with any of the 12 possible conditions were coded as 1 and those without any of the conditions were coded as 0. In contrast, the CHA_2_DS_2_-VASc includes the additional criteria: 2 points for age ≥75 years, 1 point for vascular disease (previous MI, peripheral artery disease, aortic plaque), 1 point for female sex, and 1 point for ages 65 to 74 years. CHA_2_DS_2_-VASc scores range from 0 to 9. Scores ≥2 points were considered to be high risk and 0 was considered a low risk for stroke.

The findings from an earlier study comparing the newer CHA_2_DS_2_-VASc to the older CHADS_2_ [13] found no statistically significant differences between the risk stratification results for different population groups and the associated warfarin prescribing patterns. Further, Odum, et al., [17] noted that the implementation of this risk schema warrants further and continual assessment. With these considerations in mind, we examined differences in prescribing patterns and risk stratification comparing both tools.

Warfarin prescription was the dependent variable for this study. Using the CDC’s New Ambulatory Care Drug Database System for NAMCS data, prescribed drugs were classified as warfarin or other. Six separate drugs were re-coded as the variable warfarin. These were: Jantoven, Athrombin K, Coumadin, Panwarfin, Warcef, and warfarin.

In order to identify patients at risk for bleeding, the three digit ICD-9 codes associated with GI hemorrhage were merged into a variable called *bleed risk*. All patients with a risk for GI bleeding were excluded from the study.

Statistical Package for Social Scientists (SPSS, IBM, Chicago, IL, version 23.0) was used to complete all statistical analyses and alpha was set at *p* ≤ 0.05. Bivariate contingency table analysis was conducted to establish the relationships between each of the covariates and the dependent variable. Bivariate analysis tests whether or not a statistically significant relationship exists between an outcome or dependent variable and a predictor or independent variable. Bivariate analysis is not a stratified analysis. A chi square was computed as the test statistic for differences between percentages. Multivariate logistic regression analysis, to produce adjusted measures and eliminate confounding, was also performed using SPSS (version 23.0).

The Institutional Review Board (IRB) at the researchers’ institutions recognize that the analysis of de-identified and publicly available data does not constitute human subjects research as defined in federal regulations and as such does not require IRB review. Hence, human subjects’ approval was not necessary nor sought since this was a de-identified data only study.

## Results

The study population was 7,669,844 adult AF patients. Table 1 displays the characteristics of US adults with AF. Of note, this population is more male than female, Caucasian than non-Caucasian, university graduates, and almost evenly divided by percent poverty in neighborhood. In terms of individual variables used to calculate the CHADS_2_ score, in order, hypertension was the most prevalent followed by age ≥ 75, diabetes, and congestive heart failure. The variable contributing the least to the CHADS_2_ score was history of stroke. Twelve percent of the population was at minimal risk for stroke, 35% was at moderate stroke risk, and 53% was at high stroke risk.

**Table 1:** AFIB Population Description 2010 NAMCS Data (weighted *n* = 7,669,844)

Variable	Factor	Frequency	Percent
Sex	Female	3548521	46.3
	Male	4121323	53.7
Health Insurance Status	Have Health Insurance	7428685	96.9
	Do Not Have Health Insurance	241159	3.1
Race	Caucasian	6571700	85.7
	Non-Caucasian	1098144	14.3
Poverty Percent In Patient Zip Code	<10%	3705869	48.3
	≥10%	3636363	47.4
	Unknown	327612	4.3
Education	<20% University Graduates	3003060	39.2
	≥20% University Graduates	4339172	56.6
	Unknown	327612	4.3
Geographic Locale	Rural	1288012	16.8
	Non-Rural	6205257	80.9
	Unknown	176575	2.3
Primary HCP	Yes	3128743	40.8
	No	4095339	53.4
	Unknown	445762	5.8
Warfarin	No	6297368	82.1
	Yes	1372476	17.9
Congestive Heart Failure	No	6584055	85.8
	Yes	1085789	14.2
Diabetes	No	5942040	77.5
	Yes	1727804	22.5
Hypertension	No	2512157	32.8
	Yes	5157687	67.2
History of Stroke or TIA	No	7576285	98.8
	Yes	93559	1.2
CHADS Age	<75 Years	3544600	46.2
	≥75 Years	4125244	53.8
CHADS_2_ Score^a^	Low Score	3596112	46 .9
	High Score	4073732	53.1

^a^To calculate a CHADS_2_ score 1 point is assigned to each of the variables: congestive heart failure, hypertension, ≥75 years, diabetes, and 2 points for history of stroke or TIA

Table 2 displays the results of a bivariate analysis that examined the relationship between each independent covariate by warfarin prescription status (yes or no) for patients with AF. The differences for warfarin prescription status by each of the covariates were statistically significant. Most notably, of those AF patients with a high CHADS_2_ score, 25.1% had received a warfarin prescription from their HCP.

**Table 2:** Study Covariates by Warfarin Prescription for Adults with Atrial Fibrillation NAMCS 2010 Data (weighted *n* = 7669844)

Variables and factors		Warfarin prescription status^a^	
		% Other or No Medication	% Warfarin
Sex	Female	81.2	18.8
	Male	82.9	17.1
Health Insurance Status	Have Health Insurance	82.2	17.8
	Do Not Have Health Insurance	79.6	20.4
Race	Caucasian	81.5	18.5
	Non-Caucasian	85.9	14.1
Poverty Percent In Patient Zip Code	<10%	83.9	16.1
	≥10%	79.1	20.9
Education Percent In Patient Zip Code	<20% University Graduates	77.4	22.6
	≥20% Codes University Graduates	84.4	15.6
Geographic Locale	Rural	83.3	16.7
	Non-Rural	81.6	18.4
Primary HCP	Yes	79.7	20.3
	No	82.2	17.8
	Unknown	98.3	1.7
Individual CHADS_2_ Scoring Components			
Congestive heart failure	No	84.8	15.2
	Yes	65.6	34.4
Hypertension	No	91.8	8.2
	Yes	77.4	22.6
Age	<75 Years	89.7	10.3
	≥75 Years	75.6	24.4
Diabetes	No	86.0	14.0
	Yes	68.6	31.4
History Of Stroke\TIA	No	81.9	18.1
	Yes	100.0	0.0
CHADS_2_ Score	Low Score (0–1)	92.7	7.3
	High Score (2 and higher)	74.9	25.1

^a^The differences for warfarin prescription status by each of the covariates was statistically significant (*p* < .05)

Table 3 displays the analysis conducted examining CHADS_2_ score by age range of patients with AF by whether or not the patient had a prescription for warfarin. For patients with a low CHADS_2_ score, 12.4% of those 40 – 64 years of age and 8.4% ≥ 65 years had a prescription for warfarin. For those patients with a high CHADS_2_ score, 33.0% of those 40 – 64 years of age and 24.6% ≥ 65 years had a prescription for warfarin.

**Table 3:** Warfarin Prescription by Patient Age and CHADS_2_ Score Level for Patients with Atrial Fibrillation 2010 NAMCS Data (weighted *n* = 7669844)

CHADS_2_ Score	Patient Age	Warfarin Prescription Status			
		No		Yes	
		#	%	#	%
Low Score (0–1)	18–39	80454	100.0	0	0
	40–64	1172589	87.6	165537	12.4
	≥65	1994254	91.6	183278	8.4
High Score (2 And Higher)	18–39	0	0	0	0
	40–64	158286	67.0	77884	33.0
	≥65	2891785	75.4	945777	24.6
Total	18–39	80454	100.0	0	0
	40–64	1330875	84.5	243421	15.5
	≥65	4886039	81.2	1129055	18.8

Table 4 displays the findings from the logistic regression analysis performed using *having a warfarin prescription* as the dependent variable. Seven covariates were entered into the model. The analysis yielded that patients with AF who were ≥ 40 years of age had higher odds of having a warfarin prescription if they had a high CHADS_2_ score, were Caucasian, lived in a zip code where < 20% of the population had a university education, and lived in a zip code where < 10% of the population were living in households with incomes below the federal poverty level. Further, the analysis yielded that patients with AF who were ≥ 40 years of age had lesser odds of having a warfarin prescription if they were ≥ 65 years of age, female, or had health insurance.

**Table 4:** Logistic Regression Model with Dependent Variable Warfarin Prescription for US Adults ≥40 Years with Atrial Fibrillation

Variables and factors		Adjusted odds ratios (95% CI)
Age	40–64	--^a^
	≥65	.107 (.107,.108)
Education Percent In Patient Zip Code	<20% University Graduates	1.379 (1.377,1.381)
	≥20% University Graduates	--^a^
CHADS_2_ Score	Low Score (0–1)	--^a^
	High Score (2 and Higher)	2.911 (2.906,2.915)
Sex	Female	.548 (.547,.549)
	Male	--^a^
Race	Caucasian	2.393 (2.386,2.399)
	Non-Caucasian	--^a^
Health Insurance Status	Have Health Insurance	.817 (.814,.820)
	Do Not Have Health Insurance	--^a^
Percent Poverty in Patient Zip Code	<10% Below Federal Poverty Level	1.169 (1.167,1.171)
	≥10% Below Federal Poverty Level	--^a^

^a^referent category

Table 5 displays a comparison of CHADS_2_ with CHA_2_DS_2_-VAS_C_ in terms of percent of AF patient population by risk category (high, intermediate, or low) by percent warfarin prescription and by percent difference for risk category and warfarin prescription. The comparison yielded that the 2010 AF patient population would have a larger proportion categorized with a high stroke risk (higher by 46.7%) and lower proportions with both intermediate and low stroke risk (by 112.8 and 86.4% respectively). While 25.1% of high stroke risk AF patients had a warfarin prescription using the CHADS_2_ criteria, 18.1% (28.7% fewer) had such when applying the CHA_2_DS_2_-VAS_C_ criteria.

**Table 5:** Comparison of CHADS_2_ and CHADS_2_VASC_2_ NAMCS 2010 Data

Risk level and score	CHADS_2_		CHADS_2_VASC_2_		% Difference (Relative)	
	% Risk category	% Warfarin prescription	% Risk category	% Warfarin prescription	Risk category	Warfarin prescription
High Stroke Risk (≥2 points)	53.1	25.1	85.5	18.8	46.7	28.7
Intermediate Stroke Risk (1 point)	34.8	12.2	9.7	19.9	112.8	48.0
Low Stroke Risk (0 points)	12.1	2.5	4.8	1.3	86.4	63.2

## Discussion

Patients with AF are at higher risk for stroke than patients in normal sinus rhythm. CHADS_2_ is a tool developed to risk-stratify stroke in this patient population [6]. Our study examined the associations between CHADS_2_ score levels (low and high) and warfarin prescriptions for patients with AF in order to ascertain the prevalence of prescriber adherence to 2008 anti-thrombotic therapy for AF treatment guidelines. 2010 NAMCS data were analyzed and a number of notable findings were yielded from our analyses. The most important findings are discussed below.

First, our findings suggested that only 25% of those at highest risk for ischemic stroke were prescribed warfarin. Earlier findings are mixed on this issue. For instance, Zimetbaum, et al., [26] examining claims data for adults ≥ 18 years, found that there was no difference in the prevalence of warfarin prescribing by CHADS_2_ risk level (approximately 40% by each risk level). Raji, et al., [19] examining Medicare Part D claims data found that 67.8% of the highest risk (by CHADS_2_ score) AF patients had been prescribed warfarin. Our results are clearly much lower than these two earlier studies. These other studies examined different data—claims data versus our medical record data. These two types of data could, by definition, generate different denominators. This difference provides a partial explanation for differences in the prevalence rates identified. An additional, partial explanation for our findings may include the necessity of providers balancing the benefits of appropriate anticoagulation with the risks that are associated with warfarin [27, 28]. Even though we accounted for risk of bleeding in our analysis, providers may still have made prescribing choices based on a presumed risk of bleeding events such as intracranial hemorrhage, GI bleeding, and subdural hematoma after a fall [29].

Second, our findings indicated that when stratifying by CHADS_2_ score, strong associations between age and anticoagulation therapy in the form of a prescription for warfarin were revealed for patients with AF. These prevalence rates were higher in the younger age group (40 – 64 years) than the older age group (≥65 years) regardless of CHADS_2_ score. Other studies have reported similar findings. For instance one study, [29] using clinical administrative data from Kaiser found that older age (≥85 years) AF patients had a lower prevalence of warfarin prescription. Yet another study used *Get with the Guidelines Program* data from 2001 to 2005. The researchers evaluated warfarin adherence in patients admitted for stroke who had either AF present on admission or had a history of AF. The results revealed that patients ≥65 years received warfarin less often than younger patients, and that this ratio did not improve over time. Furthermore, gender disparities were apparent as overall, women were less likely to receive warfarin [30].

In agreement with Lewis et al., [30] we found that women > 40 years of age with AF were less likely to be prescribed warfarin. Some studies have similar findings while others have found no gender differences in warfarin prescribing patterns [31, 32]. For instance, in a Swedish study [31] examining medical records of men and women ≥ 45 years of age, no consistent associations between the CHADS_2_ score and prescribed warfarin treatment were found. Another study [32] conducted in Canada found that for patients with AF, older women (≥75 years) were half as likely to be prescribed warfarin in comparison to men in the same age group (24.5% vs. 44.9%, *p* = 0.034). Since our study included women 40 years and older with AF, the population included is more representative of those at risk for stroke in contrast to women 75 years and older. Some of the differences between our findings and those of others may be accounted for by the wider age inclusion.

Finally, our findings support the claims [17] that when using the criteria of CHA_2_DS_2_-VASc, more AF patients will be classified at high risk and fewer at intermediate risk. These changes are significant since the recommendation for anticoagulant therapy is made clearer, removing a large proportion of AF patients from the intermediate category, leaving the choice of therapy up to the provider.

Based on the key findings of our study, as discussed above, opportunities for interventions are present to improve guideline adherence in alignment with risk stratification for stroke prevention. Interprofessional health care teams can provide improved medical management of stroke prevention for patients with AF. These interprofessional health care teams should be constituted of primary care providers (physicians, physician assistants, and nurse practitioners), nurses (RN, LPN), and pharmacists (PharmD, RPh). Health care teams are not a new concept [33–38] and typically such a team refers to two or more individuals, each with a specific role, working toward a common goal with concrete boundaries [35]. Frequently, health care teams work on complex tasks requiring a coordination of effort [34–38]. In many instances developing a team approach to this type of care and medical management requires a culture shift in both specific practices as well as clinical professions [34–39]. A more integrated team care approach could lead to better guideline adherence and subsequent reduction in actual strokes in patients with AF, leading to reduced morbidity and health care cost.

Some clinicians [37] have noted that there has been a “quantum leap” in the complexity of tasks required of primary care such that physicians alone are no longer able to cope with the resulting wide scope of practice. This coupled with the ongoing demand for health care cost containment [37, 39, 40] has led to the exploration of primary care teams that include physician assistants and nurse practitioners. Moreover, a demand for better health care quality and safety has provided the opportunity for pharmacists to be included in primary care teams as health care givers with beneficial skills that complement those of physicians [37–40]. There is growing evidence, around some chronic conditions such as diabetes, that interprofessional health care teams improve patient outcomes and contribute to contained health care costs [37–40].

### Limitations

There are some limitations to our study. For instance, using NAMCS data did not allow for any analysis that would detect prescriber bias. Furthermore, NAMCS data are not longitudinal and are only point-in-time single visit data. Hence, no continuity of care is captured that might provide an indication of changes over time regarding medication use or a broader overview of prescribing patterns for individual patients. Additionally, only a limited number of diagnoses are captured on the patient survey hence it is possible that not all instances of AF were identified. Another limitation is that the education status of the patients with AF were not recorded but instead the percentage of persons with a university education in the zip code was used as a proxy. Furthermore, not all contraindications to warfarin may have been identified. Also, because of the limited number of medications that can be listed on the patient data form, warfarin may have been under-reported on the medication list. Finally, different classifications of AF were not listed nor was the duration of disease.

## Conclusions

This study underscores the importance of a comprehensive assessment of risk factors associated with stroke in patients with AF and the prescribing of warfarin. This is especially true for older (those ≥ 65 years), as well as female patients. Overall, warfarin appears to be under-prescribed for patients with AF. Given the benefit of warfarin use for those most at risk for ischemic stroke, our findings suggest that warfarin should be prescribed with closer adherence to guideline recommendations.

## Abbreviations

AF: Atrial fibrillation
CDC: Center for Disease Control and Prevention
HCP: Health Care Provider
IRB: Institutional Review Board
NAMCS: National Ambulatory Care Survey
NCHS: National Center for Health Statistics
TIA: Transient Ischemic Attack
